# Propofol reduces renal ischemia/reperfusion-induced acute lung injury by stimulating sirtuin 1 and inhibiting pyroptosis

**DOI:** 10.18632/aging.202191

**Published:** 2020-12-01

**Authors:** Zhaohui Liu, Yanli Meng, Yu Miao, Lili Yu, Qiannan Yu

**Affiliations:** 1 Department of Anesthesiology, Cangzhou Central Hospital, Cangzhou, Hebei, China; 2 Department of Gastroenterology, Cangzhou Central Hospital, Cangzhou, Hebei, China; 3 Department of Neurosurgery, Cangzhou Central Hospital, Cangzhou, Hebei, China

**Keywords:** propofol, renal ischemia/reperfusion, pyroptosis, SIRT1, acute lung injury

## Abstract

The activation of pyroptosis is an important feature of renal ischemia/reperfusion (rI/R)-induced acute lung injury (ALI). Propofol, a general anesthetic, is known to inhibit inflammation in I/R-induced ALI. We investigated whether propofol could suppress pyroptosis during rI/R-induced ALI by upregulating sirtuin 1 (SIRT1). We generated an *in vivo* model of rI/R-induced ALI by applying microvascular clamps to the renal pedicles of rats for 45 min. Pathological studies revealed that rI/R provoked substantial lung injury and inflammatory cell infiltration. The rI/R stimulus markedly activated pyroptotic proteins such as NLRP3, ASC, caspase 1, interleukin-1β and interleukin-18 in the lungs, but reduced the mRNA and protein levels of SIRT1. Propofol treatment greatly inhibited rI/R-induced lung injury and pyroptosis, whereas it elevated SIRT1 expression. Treatment with the selective SIRT1 inhibitor nicotinamide reversed the protective effects of propofol during rI/R-induced ALI. Analogous defensive properties of propofol were detected *in vitro* in rat alveolar macrophages incubated with serum from the rI/R rat model. These findings indicate that propofol attenuates rI/R-induced ALI by suppressing pyroptosis, possibly by upregulating SIRT1 in the lungs.

## INTRODUCTION

Renal ischemia/reperfusion (rI/R) is an important cause of death worldwide. The risk of mortality due to rI/R is two to three times higher for those with acute lung injury (ALI) [1–3], so it is crucial to determine ways of inhibiting this complication. Treatments regulating blood glucose concentrations, blood pressure and oxidative stress have not been found to reduce deaths associated with rI/R-induced ALI [4, 5].

Augmented serum concentrations of inflammatory factors have been recognized as strong predictors of rI/R risk and ALI development [6]. Increased inflammatory protein levels activate the pyroptosis-related nucleotide oligomerization domain (NOD)-like receptor pyrin domain-containing 3 (NLRP3) inflammasome, an important stimulator of rI/R-induced ALI [2, 7]. NLRP3 induces interleukins 18 (IL-18) and 1β (IL-1β), inflammatory proteins that are important contributors to numerous inflammatory disorders [8–10]. On the other hand, NLRP3 suppresses sirtuin 1 (SIRT1), a recognized inhibitor of alveolar macrophage injury [11, 12]; thus, restoring SIRT1 levels could be a promising way to ameliorate NLRP3 inflammasome-induced alveolar macrophage activation. There is clear evidence that inhibiting the NLRP3 inflammasome reduces inflammatory responses [13], and NLRP3 inflammasome antagonists have mostly been successful in treating rI/R [14–16], but their effects on rI/R-induced ALI have not been reported.

The γ-aminobutyric acid receptor antagonist propofol, an intravenous hypnotic drug that has been used extensively to induce and maintain sedation and general anesthesia, has been found to suppress inflammation in I/R-induced ALI, and its adverse effects are insignificant [17–19]. Propofol is known to prevent alveolar macrophage activation, but the mechanism is not well understood [20]. In this study, we assessed the effects of propofol on NLRP3 inflammasome activation, SIRT1 expression, inflammatory factor release and oxidative stress in rI/R-induced ALI.

## RESULTS

### Propofol ameliorates the rI/R-induced release of pyroptotic proteins and inflammatory cytokines in NR8383 cells

**Figure 1:**
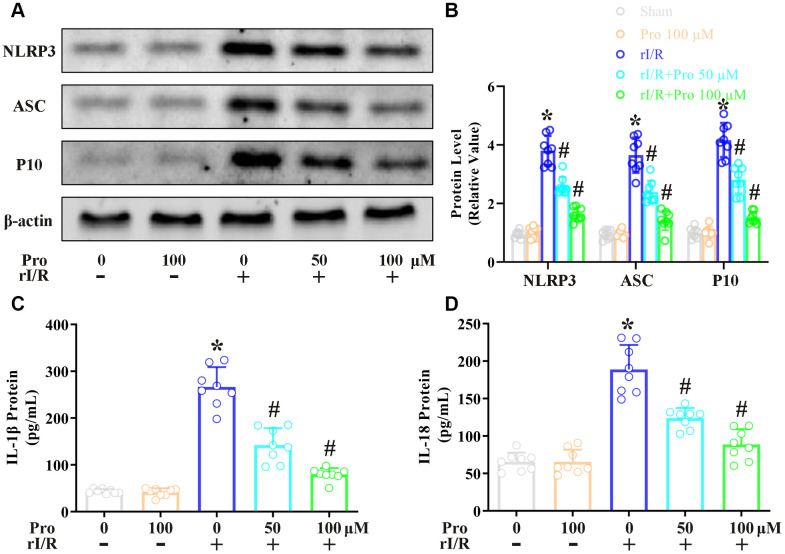
**Propofol reduces the rI/R-induced release of pyroptotic proteins and inflammatory cytokines in rat alveolar macrophages.** NR8383 cells were incubated with serum from sham or rI/R rats, with or without propofol (50 or 100 μM) for 24 h. (**A**, **B**) Western blotting was used to determine the levels of cleaved caspase 1 (P10), ASC and NLRP3. (**C**, **D**) ELISAs were used to determine the protein levels of IL-1β and IL-18 (*P < 0.05 vs. sham; ^#^*P* < 0.05 vs. rI/R).

In this study, we first examined the effects of propofol on the rI/R-induced activation of pyroptotic proteins. NR8383 rat alveolar macrophages were treated with serum from a rat model of rI/R, with or without propofol treatment. Then, Western blotting was performed to measure the protein levels of cleaved caspase 1 (P10), apoptosis-associated speck-like protein containing a CARD (ASC) and NLRP3. As indicated in Figure 1A, 1B, stimulation with serum from rI/R rats increased P10 levels 4.2-fold, ASC levels 3.6-fold, and NLRP3 levels 3.7-fold. However, the administration of 50 and 100 μM propofol reduced the levels of these three proteins dose-dependently in rI/R serum-treated cells (*P*<0.05, Figure 1A, 1B). Specifically, with the respective doses of propofol, P10 levels were reduced to 2.8- and 1.6-fold of control levels, ASC levels were reduced to 2.4- and 1.3-fold of control levels, and NLRP3 levels were reduced to 2.7- and 1.7-fold of control levels (*P*<0.05, Figure 1A, 1B).

Next, we investigated whether the inactivation of pyroptotic proteins by propofol inhibited the secretion of IL-18 and IL-1β from NR8383 cells. An enzyme-linked immunosorbent assay (ELISA) indicated that IL-1β levels were 41.5 pg/mL in control cells, but increased to 266.6 pg/mL in cells stimulated with serum from rI/R rats for 24 h (Figure 1C, 1D). The administration of 50 and 100 μM propofol to rI/R serum-treated cells reduced IL-1β levels to 142.3 and 79.5 pg/mL, respectively (*P*<0.05, Figure 1C). Likewise, IL-18 levels were 65.5 pg/mL in control cells and increased to 188.9 pg/mL in cells incubated with serum from rI/R rats for 24 h; however, the two dosages of propofol reduced IL-18 levels to 123.9 and 88.6 pg/mL, respectively (*P*<0.05, Figure 1D). These results demonstrated that propofol exerted anti-inflammatory effects by reducing the levels of pyroptotic proteins and inflammatory cytokines in NR8383 cells.

### Propofol inhibits the rI/R-induced downregulation of SIRT1 in NR8383 cells

**Figure 2:**
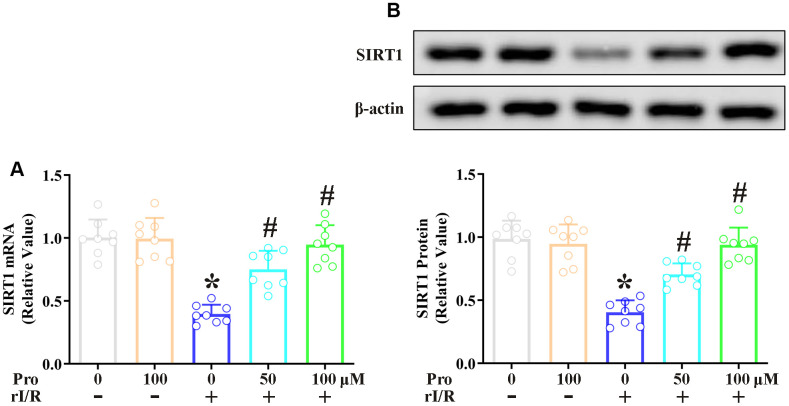
**Propofol suppresses the rI/R-induced downregulation of SIRT1 in rat alveolar macrophages.** NR8383 cells were incubated with serum from sham or rI/R rats, with or without propofol (50 or 100 μM) for 24 h. (**A**) Real-time PCR was used to measure *SIRT1* mRNA levels. (**B**) Western blotting was used to measure SIRT1 protein levels (*P < 0.05 vs. sham; ^#^*P* < 0.05 vs. rI/R).

Next, we explored the effects of propofol on the mRNA and protein levels of SIRT1 in NR8383 cells. Real-time PCR analysis revealed that the stimulation of NR8383 cells with serum from rI/R rats reduced *SIRT1* mRNA levels to 39% of control levels, whereas further treatment with 50 and 100 μM propofol restored *SIRT1* mRNA levels to 75% and 94% of control levels, respectively (*P*<0.05, Figure 2A). Furthermore, Western blotting indicated that the stimulation of NR8383 cells with serum from rI/R rats reduced SIRT1 protein levels to 40% of control levels, whereas the two dosages of propofol increased SIRT1 protein levels in rI/R serum-treated cells to nearly 72% and 95% of control levels, respectively (*P*<0.05, Figure 2B).

### The inhibition of SIRT1 reverses the anti-pyroptotic effects of propofol in rI/R-induced NR8383 cells

**Figure 3:**
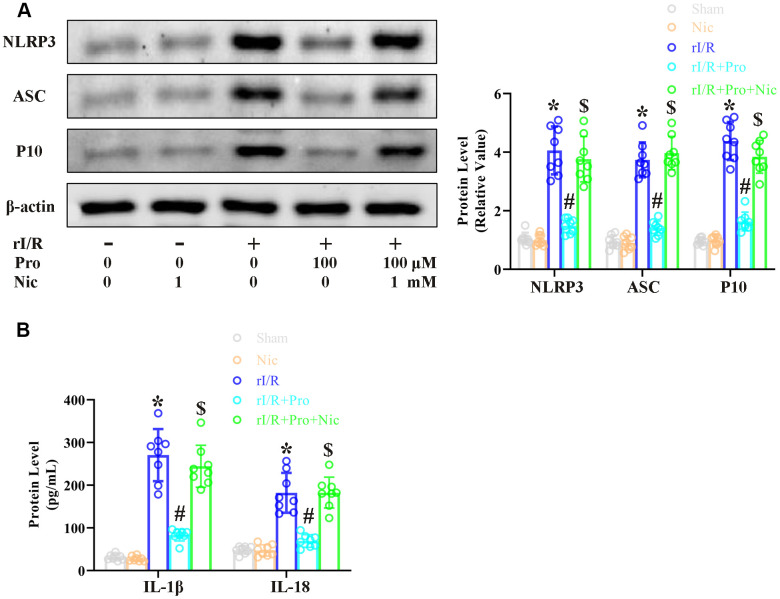
**The SIRT1 inhibitor nicotinamide prevents propofol from reducing rI/R-induced pyroptosis in rat alveolar macrophages.** NR8383 cells were incubated with serum from sham or rI/R rats, with or without propofol (100 μM) and/or nicotinamide (1 mM) for 24 h. (**A**) Western blotting was used to measure the protein levels of cleaved caspase 1 (P10), ASC and NLRP3. (**B**) ELISAs were used to determine the protein levels of IL-18 and IL-1β (*P < 0.05 vs. sham; ^#^*P* < 0.05 vs. rI/R; ^$^*P* < 0.05 vs. rI/R + propofol).

To complete our *in vitro* study, we used the SIRT1 inhibitor nicotinamide to evaluate whether the effects of propofol depended on SIRT1 in NR8383 cells. The cells were incubated with serum from rI/R rats, with or without propofol (100 μM) and/or nicotinamide (1 mM) for 24 h. Western blotting indicated that NLRP3 protein levels increased to 4.1-fold of control levels in the rI/R group, decreased to 1.4-fold of control levels in the rI/R + propofol group, and increased again to 3.8-fold of control levels in the rI/R + propofol + nicotinamide group (*P*<0.05, Figure 3A). P10 and ASC levels respectively increased to 4.4- and 3.7-fold of control levels in the rI/R group, decreased to 1.4- and 1.6-fold of control levels in the rI/R + propofol group, and increased to 3.8- and 3.9-fold of control levels in the rI/R + propofol + nicotinamide group (*P*<0.05, Figure 3A). Furthermore, ELISAs indicated that suppressing SIRT1 using nicotinamide reversed the effects of propofol on IL-18 and IL-1β levels (*P*<0.05, Figure 3B). These results indicated that SIRT1 was a crucial facilitator of the anti-pyroptotic effects of propofol in rI/R-induced NR8383 cells.

### Propofol ameliorates the morphological characteristics of rI/R-induced ALI in rats

**Figure 4:**
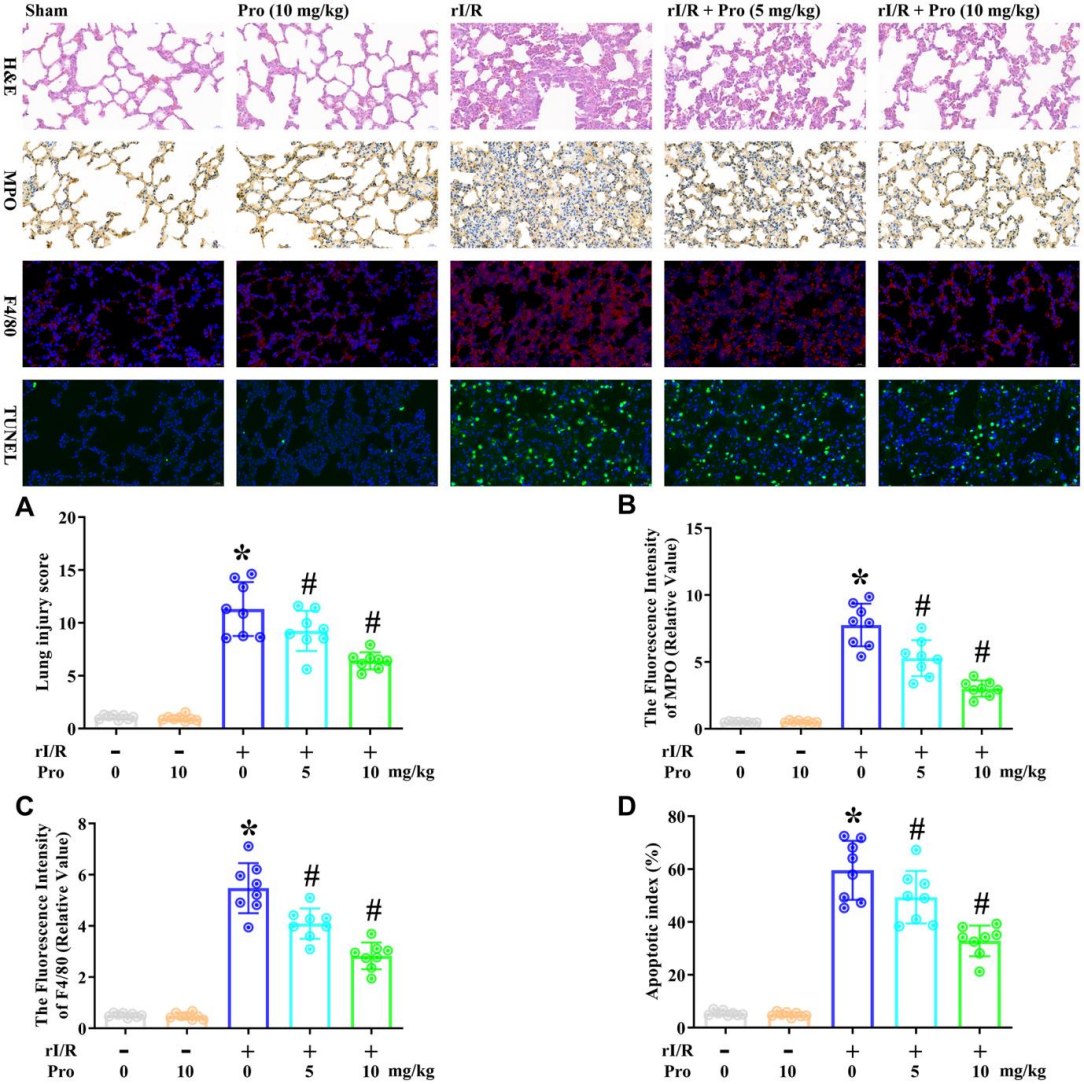
**Propofol ameliorates the morphological characteristics of rI/R-induced ALI in rats.** Rats were subjected to rI/R, with or without propofol (5 or 10 mg/kg) treatment for 24 h. (**A**) H&E analysis was performed, and the lung injury score was determined. (**B**) Immunohistochemistry was used to measure the fluorescence intensity of myeloperoxidase. (**C**) Immunofluorescence analysis was used to measure the fluorescence intensity of F4/80. (**D**) TUNEL analysis was used to measure the apoptotic index (%) (*P < 0.05 vs. sham; ^#^*P* < 0.05 vs. rI/R).

Next, we examined the effects of propofol on the morphological characteristics of rI/R-induced ALI in rats. We obtained lung tissues from our rat model of rI/R-induced ALI, and used these tissues for hematoxylin and eosin (H&E) staining, immunohistochemical analysis of myeloperoxidase, immunofluorescence analysis of F4/80, and terminal deoxynucleotidyl transferase dUTP nick end labeling (TUNEL) analysis. Rats subjected to a sham operation were used as controls. Compared with the sham treatment, rI/R increased the lung injury score 10.5-fold, the fluorescence intensity of myeloperoxidase 13.9-fold, the fluorescence intensity of F4/80 10.7-fold, and the apoptotic index (%) 10.9-fold (Figure 4). Treatment of rI/R rats with propofol (5 or 10 mg/kg) remarkably and dose-dependently reduced these four measures (*P*<0.05, Figure 4A–4D). Specifically, with the respective doses of propofol, the lung injury scores were reduced to 8.6- and 5.9-fold of sham levels, the fluorescence intensity levels of myeloperoxidase were reduced to 9.4- and 5.4-fold of sham levels, the fluorescence intensity levels of F4/80 were reduced to 8.0- and 5.5-fold of sham levels, and the apoptotic indexes (%) were reduced to 9.0- and 6.0-fold of sham levels (*P*<0.05, Figure 4A–4D). These results demonstrated that propofol ameliorated the morphological characteristics of rI/R-induced ALI in rats.

### Propofol ameliorates the rI/R-induced release of pyroptotic proteins and inflammatory cytokines in the lungs of rats

**Figure 5:**
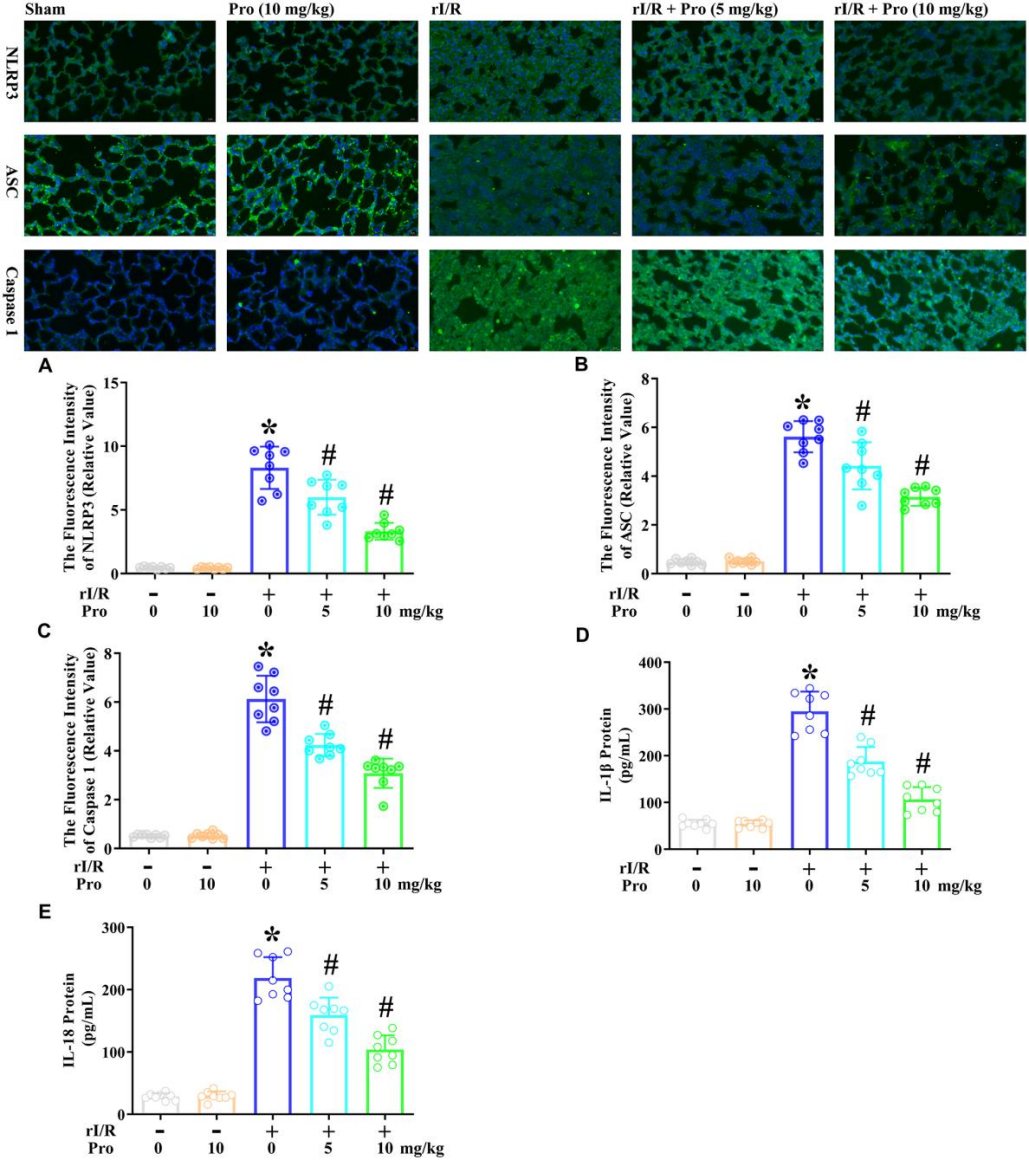
**Propofol suppresses the rI/R-induced release of pyroptotic proteins and inflammatory cytokines in the lungs of rats.** Rats were subjected to rI/R, with or without propofol (5 or 10 mg/kg) treatment for 24 h. (**A**–**C**) Immunofluorescence analyses were used to measure the fluorescence intensity levels of NLRP3, ASC and caspase 1. (**D**, **E**) ELISAs were used to measure the protein levels of IL-1β and IL-18 (*P < 0.05 vs. sham; ^#^*P* < 0.05 vs. rI/R).

We then examined the effects of propofol on the rI/R-induced activation of pyroptotic proteins. We performed immunofluorescence analyses to evaluate the protein levels of cleaved caspase 1 (P10), ASC and NLRP3 in lung tissues from rats subjected to rI/R, with or without propofol treatment. As indicated in Figure 5A–5C, rI/R enhanced the fluorescence intensity of NLRP316.6-fold, the fluorescence intensity of ASC 11.2-fold, and the fluorescence intensity of caspase 1 12.2-fold compared with the sham operation. Propofol treatment at 5 and 10 mg/kg dose-dependently reduced the levels of these three proteins in the lungs of rI/R rats (*P*<0.05, Figure 5A–5C). Specifically, with the respective doses of propofol, the fluorescence intensity levels of NLRP3 were reduced to 12.0- and 6.6-fold of sham levels, the fluorescence intensity levels of ASC were reduced to 8.8- and 6.3-fold of sham levels, and the fluorescence intensity levels of caspase 1 were reduced to 8.5- and 6.2-fold of sham levels (*P*<0.05, Figure 5A–5C).

We also explored whether the propofol-induced inactivation of pyroptotic proteins inhibited IL-18 and IL-1β secretion in the lungs of rats. An ELISA indicated that IL-1β levels in the lungs were 54.8 pg/mL in the sham group, but increased to 294.9 pg/mL after 24 h of rI/R (Figure 5D, 5E). Treatment of rI/R rats with propofol at 5 and 10 mg/kg reduced IL-1β levels to 187.4 and 106.4 pg/mL, respectively (*P*<0.05, Figure 5D). Likewise, IL-18 levels were 28.3 pg/mL in the sham group, increased to 218.6 pg/mL in the rI/R group, and decreased to 159.2 and 104.0 pg/mL in the rI/R + propofol (5 and 10 mg/kg) groups, respectively (*P*<0.05, Figure 5E). These results demonstrated that propofol exerted anti-inflammatory effects by inactivating pyroptotic proteins and inflammatory cytokines in the lungs of rats.

### Propofol prevents the rI/R-induced downregulation of SIRT1 in the lungs of rats

**Figure 6:**
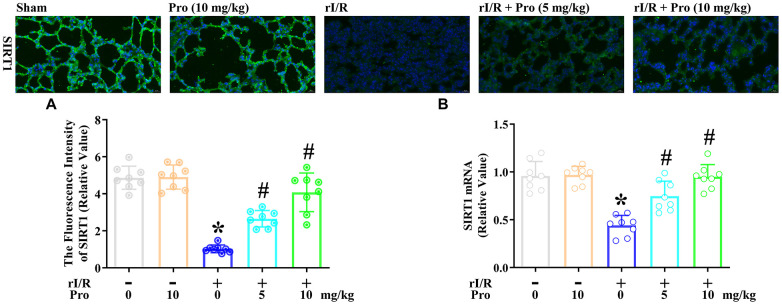
**Propofol inhibits the rI/R-induced downregulation of SIRT1 in the lungs of rats.** Rats were subjected to rI/R, with or without propofol (5 or 10 mg/kg) treatment for 24 h. (**A**) Immunofluorescence analysis was used to measure the fluorescence intensity of SIRT1. (**B**) Real-time PCR was used to measure the mRNA expression of *SIRT1* (*P < 0.05 vs. sham; ^#^*P* < 0.05 vs. rI/R).

Subsequently, we assessed the effects of propofol on the mRNA and protein levels of SIRT1 in the lungs of rI/R rats. An immunofluorescence analysis indicated that rI/R reduced the fluorescence intensity of SIRT1 to 21% of the sham level, whereas further treatment with propofol at 5 and 10 mg/kg increased it to nearly 55% and 84% of the sham level, respectively (*P*<0.05, Figure 6A). Furthermore, real-time PCR analysis indicated that rI/R reduced the *SIRT1* mRNA level to about 46% of the sham level, while the respective doses of propofol increased *SIRT1* mRNA levels in rI/R rats to 78% and 99% of the sham level (*P*<0.05, Figure 6B).

### The inhibition of SIRT1 reverses the anti-pyroptotic effects of propofol in the lungs of rI/R rats

**Figure 7:**
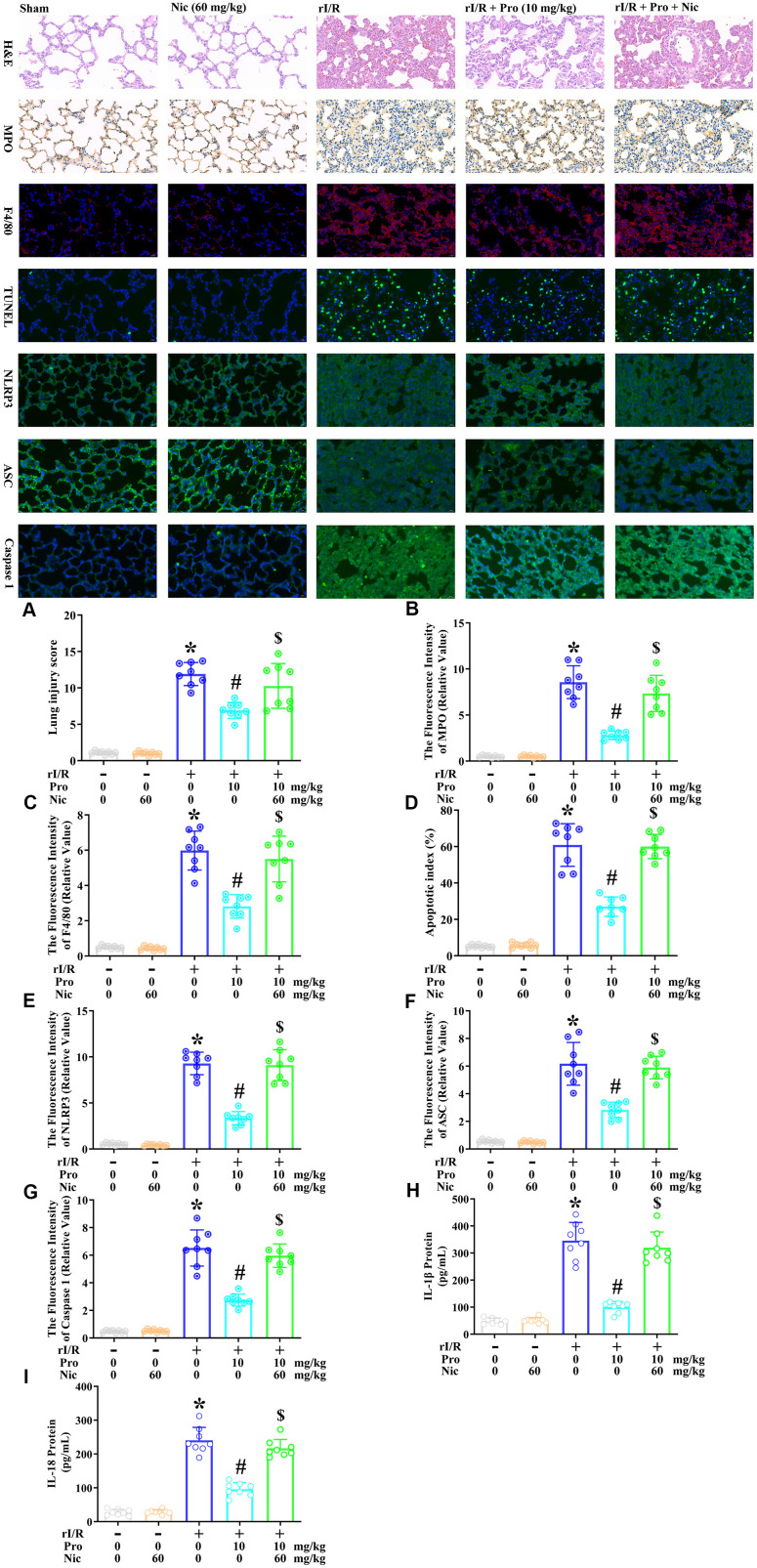
**The inhibition of SIRT1 prevents propofol from ameliorating the morphological characteristics of ALI and suppressing pyroptosis in the lungs of rI/R rats.** Rats were subjected to rI/R, with or without propofol (10 mg/kg) and/or nicotinamide (60 mg/kg) treatment for 24 h. (**A**) H and E analysis was performed, and the lung injury score was determined. (**B**) Immunohistochemistry was used to measure the fluorescence intensity of myeloperoxidase. (**C**) Immunofluorescence analysis was used to measure the fluorescence intensity of F4/80. (**D**) TUNEL analysis was used to measure the apoptotic index (%). (**E**–**G**) Immunofluorescence analyses were used to measure the fluorescence intensity levels of NLRP3, ASC and caspase 1. (**H**, **I**) ELISAs were used to measure the protein levels of IL-1β and IL-18 (*P < 0.05 vs. sham; ^#^*P* < 0.05 vs. rI/R; ^$^*P* < 0.05 vs. rI/R + propofol).

Lastly, we used the SIRT1 inhibitor nicotinamide to evaluate whether the effects of propofol depended on SIRT1 in the lungs of rats. Rats were subjected to rI/R with or without propofol (10 mg/kg) and/or nicotinamide (60 mg/kg) treatment for 24 h. Then, H&E staining, immunohistochemical analysis of myeloperoxidase, immunofluorescence analysis of F4/80, TUNEL analysis, and immunofluorescence analyses of NLRP3, ASC and caspase 1 were performed on their lung tissues. As shown in Figure 7A–7G, rI/R enhanced the lung injury score 10.9-fold, the fluorescence intensity of myeloperoxidase 17.1-fold, the fluorescence intensity of F4/80 11.8-fold, the apoptotic index (%) 11.7-fold, the fluorescence intensity of NLRP3 16.9-fold, the fluorescence intensity of ASC 11.2-fold, and the fluorescence intensity of caspase 1 13.6-fold compared with the sham operation. Propofol treatment of rI/R rats reduced the lung injury score to 6.3-fold of the sham level, the fluorescence intensity of myeloperoxidase to 5.6-fold of the sham level, the fluorescence intensity of F4/80 to 5.6-fold of the sham level, the apoptotic index (%) to 5.2-fold of the sham level, the fluorescence intensity of NLRP3 to 6.1-fold of the sham level, the fluorescence intensity of ASC to 5.2-fold of the sham level, and the fluorescence intensity of caspase 1 to 5.7-fold of the sham level. However, when nicotinamide was used to inhibit SIRT1, the effects of propofol were suppressed, such that the lung injury score increased to 9.4-fold of the sham level, the fluorescence intensity of myeloperoxidase increased to 14.6-fold of the sham level, the fluorescence intensity of F4/80 increased to 10.9-fold of the sham level, the apoptotic index (%) increased to 11.5-fold of the sham level, the fluorescence intensity of NLRP3 increased to 16.6-fold of the sham level, the fluorescence intensity of ASC increased to 10.7-fold of the sham level, and the fluorescence intensity of caspase 1 increased to 12.4-fold of the sham level (*P*<0.05, Figure 7A–7G). Moreover, ELISAs indicated that nicotinamide reversed the effects of propofol on IL-1β and IL-18 secretion in the lungs (*P*<0.05, Figure 7H, 7I). These results indicated that SIRT1 was a crucial facilitator of the anti-pyroptotic effects of propofol in the lungs of rI/R rats.

## DISCUSSION

In this investigation, we first stimulated NR8383 rat alveolar macrophages with serum from rI/R rats and assessed the effects of propofol on numerous indicators of rI/R injury, including the activation of the NLRP3 inflammasome, the induction of various other pyroptosis-related inflammatory molecules and the suppression of SIRT1. Our results revealed that, at clinically relevant doses, propofol hindered each of these features of pyroptosis. IL-1β and IL-18 are crucial activation elements of the NLRP3 inflammasome during pyroptosis [21], and their production is known to be activated by I/R and sepsis but suppressed by propofol [22–24]. Accordingly, the present results indicated that propofol could inhibit the activation of IL-1β and IL-18 in rat alveolar macrophages and lung tissues during rI/R.

Inhibiting the activation of the pyroptosis-related NLRP3 inflammasome is considered a key treatment strategy for several chronic inflammatory disorders; however, a harmless and dependable therapy remains to be identified. Using lung tissues from a rat model of rI/R-induced ALI, we confirmed our *in vitro* findings that propofol could attenuate pyroptosis by inhibiting the expression of pyroptotic proteins and enhancing the expression of SIRT1 [25, 26]. These results extended previous findings that propofol could stop the progression of rI/R by reducing the levels of pyroptotic proteins [27].

Notably, our study also revealed that SIRT1 was a crucial conveyer of the beneficial effects of propofol, as demonstrated by the results of inhibiting SIRT1 using nicotinamide. Propofol has been reported to activate SIRT1 in hepatic I/R tissues and human umbilical vein endothelial cells [28, 29]. We found that propofol enhanced SIRT1 expression in NR8383 cells, suggesting that propofol could be used to inhibit pulmonary disorders by inducing SIRT1. Further *in vitro* and *in vivo* research is needed to determine the mechanisms whereby propofol exerts these pro-SIRT1, anti-NLRP3 and anti-pyroptotic effects during rI/R-induced ALI.

## MATERIALS AND METHODS

### Cell culture and treatment

NR8383 cells were obtained from the cell bank of the Shanghai Institute of Cell Biology, Chinese Academy of Sciences (Shanghai, China). The cells were grown in Dulbecco’s modified Eagle’s medium with 10% fetal bovine serum at 37° C in a humidified incubator (5% CO_2_). The cells were treated with serum from rI/R rats, with or without propofol (50 or 100 μM) and/or nicotinamide (1 mM).

### rI/R model establishment and drug treatment

Sprague-Dawley rats (220-250 g) were obtained from the Experimental Animal Center of Hebei Medical University (Hebei, China), and were housed in a rat facility under representative experimental conditions for seven days before the studies began. All the experiments were conducted according to the National Institutes of Health Guidelines for the Care and Use of Experimental Animals, and the animal procedures were approved by Cangzhou Central Hospital. The rats received water and ordinary chow *ad libitum*.

An *in vivo* model of rI/R-induced ALI was generated by applying microvascular clamps to the renal pedicles of rats for 45 min. The effects of propofol on rI/R-induced ALI were evaluated using the following five randomly assigned groups of rats (n = 8/group): the sham group (subjected to the same operation, without the clamping of the renal pedicles), the propofol (10 mg/kg) group, the rI/R group, the rI/R + propofol (5 mg/kg) group and the rI/R + propofol (10 mg/kg) group. The effects of the SIRT1 inhibitor nicotinamide were assessed using the following five randomly assigned groups of rats (n = 8/group): the sham group, the nicotinamide (60 mg/kg) group, the rI/R group, the rI/R + propofol (10 mg/kg) group and the rI/R + propofol (10 mg/kg) + nicotinamide (60 mg/kg) group. The treatment process was performed as previously described by Liu et al [2, 3]. The rats were sacrificed via intraperitoneal administration of 120 mg/kg sodium thiopental, 24 h after rI/R with or without the additional treatments. Lung samples were obtained for pathological, molecular and biological studies.

### H&E analysis

H&E staining was performed at 37° C to prepare the lung samples for morphological examination. The degree of lung injury was graded histologically using the Murakami method by an investigator blinded to the treatment groups. Twenty-four areas of the lung parenchyma were scored separately for edema, congestion, hemorrhage and inflammation on a scale from 0 to 4 (0, absent and appears normal; 1, light; 2, moderate; 3, strong; and 4, intense). Image-Pro Plus software 4.5 (Media Cybernetics, Silver Spring, MD, USA) was used for image analysis.

### Immunohistochemistry

In brief, lung tissues from the aforementioned groups of rats were fixed with paraformaldehyde (4%) at 37° C for 10 min. Subsequently, 0.1% Triton X-100 in Tris-buffered saline with Tween 20 (TBST) was used to permeabilize the lung sections for 15 min. Then, TBST containing 2.5% fetal bovine serum and 5% bovine serum albumin was used to block the lung sections, and an anti-myeloperoxidase primary antibody (AF7494, Beyotime, 1:50) was applied at 4° C overnight. The lung sections were then washed three times and treated with a secondary antibody (A0181, Beyotime, 1:100) at 37° C for 1 h.

### TUNEL analysis

A TUNEL analysis was performed using a cell death assay kit to assess lung apoptosis, as described previously. TUNEL-positive nuclei exhibited green fluorescence, while nuclei from TUNEL-negative cells exhibited blue fluorescence. The degree of apoptosis was calculated as the quantity of apoptotic cells/the whole number of cells measured×100%.

### Immunofluorescence analysis

Lung sections from the aforementioned groups of rats were washed three times with phosphate-buffered saline and then treated with primary antibodies against F4/80, NLRP3, ASC, caspase 1 and SIRT1 at 4° C overnight. Subsequently, the tissues were probed with a secondary antibody (A0423, Beyotime, 1:500), washed with phosphate-buffered saline and sealed with glycerin (95%). A fluorescence microscope was used to visualize the fluorescence indicators.

### Real-time PCR analysis

An RNeasy Micro Kit was used to isolate total RNA from NR8383 cells and rat lung tissues according to the manufacturer’s directions (QIAGEN, UK). A Nanodrop spectrophotometer was used to measure RNA levels. Complementary DNA was obtained from 1 μg of RNA using the iScript^TM^ Reverse Transcription Supermix. Then, rat *SIRT1* mRNA transcripts were amplified with a SYBR Green-based real-time PCR assay (Invitrogen) on an ABI 7500 platform. *GAPDH* was used to normalize the data according to the 2^-ΔΔCt^ method.

### Western blotting assay

Radioimmunoprecipitation assay buffer with protease and phosphatase inhibitors was used to lyse the NR8383 cells. Sodium dodecyl sulfate-polyacrylamide gel electrophoresis (10%) was used to separate the proteins from 20 μg of cell lysate, and then the proteins were transferred to polyvinylidene fluoride membranes. Primary antibodies against NLRP3, ASC, P10, SIRT1 and β-actin (Beyotime, Wuhan, China) were used to blot the membranes, and then the corresponding secondary antibodies were applied (horseradish peroxidase-labeled antibody; Beyotime). Pierce^TM^ ECL Plus Western blot substrate was used to visualize the immunoblots.

### ELISA analysis

The supernatants of NR8383 cells and homogenates of rat lung tissues were evaluated for their concentrations of IL-18 and IL-1β. ELISA kits were acquired from Boster Biological Technology Co. Ltd. and used according to the manufacturer’s directions. The results were obtained using spectrometry on a 96-plate reader.

### Statistical analysis

The findings are presented as the mean ± standard derivation. Statistical analyses were carried out using one-way analysis of variance followed by Bonferroni’s post-test comparisons in GraphPad Prism 8. *P* values <0.05 were considered statistically significant.

## References

[1] Hayase N, Doi K, Hiruma T, Matsuura R, Hamasaki Y, Noiri E, Nangaku M, Morimura N. Recombinant thrombomodulin prevents acute lung injury induced by renal ischemia-reperfusion injury. Sci Rep. 2020; 10:289. 10.1038/s41598-019-57205-0 31937858 PMC6959219

[2] Liu Z, Qu M, Yu L, Song P, Chang Y. Artesunate inhibits renal ischemia-reperfusion-mediated remote lung inflammation through attenuating ROS-induced activation of NLRP3 inflammasome. Inflammation. 2018; 41:1546–56. 10.1007/s10753-018-0801-z 29730819

[3] Liu Z, Zhang J, Li S, Jiang J. Artesunate inhibits renal ischemia reperfusion-stimulated lung inflammation in rats by activating HO-1 pathway. Inflammation. 2018; 41:114–21. 10.1007/s10753-017-0669-3 28921399

[4] Hassoun HT, Lie ML, Grigoryev DN, Liu M, Tuder RM, Rabb H. Kidney ischemia-reperfusion injury induces caspase-dependent pulmonary apoptosis. Am J Physiol Renal Physiol. 2009; 297:F125–37. 10.1152/ajprenal.90666.2008 19403643 PMC2711715

[5] White LE, Cui Y, Shelak CM, Lie ML, Hassoun HT. Lung endothelial cell apoptosis during ischemic acute kidney injury. Shock. 2012; 38:320–27. 10.1097/SHK.0b013e31826359d0 22777112

[6] Stapleton RD, Dixon AE, Parsons PE, Ware LB, Suratt BT, and NHLBI Acute Respiratory Distress Syndrome Network. The association between BMI and plasma cytokine levels in patients with acute lung injury. Chest. 2010; 138:568–77. 10.1378/chest.10-0014 20435656 PMC2940070

[7] Ma J, Chen Q, Li J, Zhao H, Mi E, Chen Y, Yi B, Ning J, Ma D, Lu K, Gu J. Dexmedetomidine-mediated prevention of renal ischemia-reperfusion injury depends in part on cholinergic anti-inflammatory mechanisms. Anesth Analg. 2020; 130:1054–62. 10.1213/ANE.0000000000003820 30346356

[8] Pirzada RH, Javaid N, Choi S. The roles of the NLRP3 inflammasome in neurodegenerative and metabolic diseases and in relevant advanced therapeutic interventions. Genes (Basel). 2020; 11:131. 10.3390/genes11020131 32012695 PMC7074480

[9] Danielski LG, Giustina AD, Bonfante S, Barichello T, Petronilho F. The NLRP3 inflammasome and its role in sepsis development. Inflammation. 2020; 43:24–31. 10.1007/s10753-019-01124-9 31741197

[10] Zhang X, Xu A, Lv J, Zhang Q, Ran Y, Wei C, Wu J. Development of small molecule inhibitors targeting NLRP3 inflammasome pathway for inflammatory diseases. Eur J Med Chem. 2020; 185:111822. 10.1016/j.ejmech.2019.111822 31699536

[11] Imperatore F, Maurizio J, Vargas Aguilar S, Busch CJ, Favret J, Kowenz-Leutz E, Cathou W, Gentek R, Perrin P, Leutz A, Berruyer C, Sieweke MH. SIRT1 regulates macrophage self-renewal. EMBO J. 2017; 36:2353–72. 10.15252/embj.201695737 28701484 PMC5556267

[12] Li Y, Yang X, He Y, Wang W, Zhang J, Zhang W, Jing T, Wang B, Lin R. Negative regulation of NLRP3 inflammasome by SIRT1 in vascular endothelial cells. Immunobiology. 2017; 222:552–61. 10.1016/j.imbio.2016.11.002 27908642

[13] Guo Z, Yu S, Chen X, Ye R, Zhu W, Liu X. NLRP3 is involved in ischemia/reperfusion injury. CNS Neurol Disord Drug Targets. 2016; 15:699–712. 10.2174/1871527315666160321111829 26996163

[14] Tang TT, Lv LL, Pan MM, Wen Y, Wang B, Li ZL, Wu M, Wang FM, Crowley SD, Liu BC. Hydroxychloroquine attenuates renal ischemia/reperfusion injury by inhibiting cathepsin mediated NLRP3 inflammasome activation. Cell Death Dis. 2018; 9:351. 10.1038/s41419-018-0378-3 29500339 PMC5834539

[15] Wen Y, Liu YR, Tang TT, Pan MM, Xu SC, Ma KL, Lv LL, Liu H, Liu BC. mROS-TXNIP axis activates NLRP3 inflammasome to mediate renal injury during ischemic AKI. Int J Biochem Cell Biol. 2018; 98:43–53. 10.1016/j.biocel.2018.02.015 29477360

[16] Liu JJ, Lu L, Hu FQ, Yuan H, Xu Q, Qin YF, Gong JH. Methylene blue attenuates renal ischemia-reperfusion injury by negative regulation of NLRP3 signaling pathway. Eur Rev Med Pharmacol Sci. 2018; 22:2847–53. 10.26355/eurrev_201805_14986 29771438

[17] Zhao W, Zhou S, Yao W, Gan X, Su G, Yuan D, Hei Z. Propofol prevents lung injury after intestinal ischemia-reperfusion by inhibiting the interaction between mast cell activation and oxidative stress. Life Sci. 2014; 108:80–87. 10.1016/j.lfs.2014.05.009 24878149

[18] Vasileiou I, Kalimeris K, Nomikos T, Xanthopoulou MN, Perrea D, Agrogiannis G, Nakos G, Kostopanagiotou G. Propofol prevents lung injury following intestinal ischemia-reperfusion. J Surg Res. 2012; 172:146–52. 10.1016/j.jss.2010.07.034 20855084

[19] Balyasnikova IV, Visintine DJ, Gunnerson HB, Paisansathan C, Baughman VL, Minshall RD, Danilov SM. Propofol attenuates lung endothelial injury induced by ischemia-reperfusion and oxidative stress. Anesth Analg. 2005; 100:929–36. 10.1213/01.ANE.0000147707.49192.88 15781500

[20] Kotani N, Hashimoto H, Sessler DI, Yasuda T, Ebina T, Muraoka M, Matsuki A. Expression of genes for proinflammatory cytokines in alveolar macrophages during propofol and isoflurane anesthesia. Anesth Analg. 1999; 89:1250–56. 10553845

[21] Yang H, Antoine DJ, Andersson U, Tracey KJ. The many faces of HMGB1: molecular structure-functional activity in inflammation, apoptosis, and chemotaxis. J Leukoc Biol. 2013; 93:865–73. 10.1189/jlb.1212662 23446148 PMC4051189

[22] Li YM, Sun JG, Hu LH, Ma XC, Zhou G, Huang XZ. Propofol-mediated cardioprotection dependent of microRNA-451/HMGB1 against myocardial ischemia-reperfusion injury. J Cell Physiol. 2019; 234:23289–301. 10.1002/jcp.28897 31188485

[23] Feng Z, Wang JW, Wang Y, Dong WW, Xu ZF. Propofol protects lung endothelial barrier function by suppression of high-mobility group box 1 (HMGB1) release and mitochondrial oxidative damage catalyzed by HMGB1. Med Sci Monit. 2019; 25:3199–211. 10.12659/MSM.915417 31040263 PMC6507496

[24] Wang X, Liu C, Wang G. Propofol protects rats and human alveolar epithelial cells against lipopolysaccharide-induced acute lung injury via inhibiting HMGB1 expression. Inflammation. 2016; 39:1004–16. 10.1007/s10753-016-0330-6 26956470

[25] Kotani N, Hashimoto H, Sessler DI, Kikuchi A, Suzuki A, Takahashi S, Muraoka M, Matsuki A. Intraoperative modulation of alveolar macrophage function during isoflurane and propofol anesthesia. Anesthesiology. 1998; 89:1125–32. 10.1097/00000542-199811000-00012 9822000

[26] Kalimeris K, Christodoulaki K, Karakitsos P, Batistatou A, Lekka M, Bai M, Kitsiouli E, Nakos G, Kostopanagiotou G. Influence of propofol and volatile anaesthetics on the inflammatory response in the ventilated lung. Acta Anaesthesiol Scand. 2011; 55:740–48. 10.1111/j.1399-6576.2011.02461.x 21615348

[27] Wei Q, Zhao J, Zhou X, Yu L, Liu Z, Chang Y. Propofol can suppress renal ischemia-reperfusion injury through the activation of PI3K/AKT/mTOR signal pathway. Gene. 2019; 708:14–20. 10.1016/j.gene.2019.05.023 31082504

[28] Liu Y, Du X, Zhang S, Liu X, Xu G. Propofol alleviates hepatic ischemia/reperfusion injury via the activation of the Sirt1 pathway. Int J Clin Exp Pathol. 2017; 10:10959–68. 31966440 PMC6965884

[29] Wang J, Qi J, Wu Q, Jiang H, Yin Y, Huan Y, Zhao Y, Zhu M. Propofol attenuates high glucose-induced P66shc expression in human umbilical vein endothelial cells through Sirt1. Acta Biochim Biophys Sin (Shanghai). 2019; 51:197–203. 10.1093/abbs/gmy167 30590376

